# Digital Imaging and Artificial Intelligence in Infantile Hemangioma: A Systematic Literature Review

**DOI:** 10.3390/biomimetics9110663

**Published:** 2024-11-01

**Authors:** Nour Mohamed, Tamer Rabie

**Affiliations:** 1 Department of Electrical Engineering, College of Engineering, University of Sharjah, Sharjah P.O. Box 27272, United Arab Emirates; nour.mohamed@sharjah.ac.ae; 2 Department of Computer Engineering, College of Computing & Informatics, University of Sharjah, Sharjah P.O. Box 27272, United Arab Emirates

**Keywords:** artificial intelligence, infantile hemangiomas, detection, lesions, segmentation

## Abstract

Infantile hemangioma (IH) is a vascular anomaly observed in newborns, with potential severe complications if left undetected. Consequently, researchers have turned to artificial intelligence (AI) and digital imaging (DI) methods for detection, segmentation, and assessing the treatment response in IH cases. This paper conducts a systematic literature review (SLR) following the Kitchenham framework to scrutinize the utilization of AI and digital imaging techniques in IH applications. A total of 21 research articles spanning from 2014 to April 2024 were carefully selected and analyzed to address four key research questions: the issues solved in IH using AI and DI, the most-used AI and DI techniques, the best-performing technique in detecting IH, and the limitations and future directions in the various fields of IH. After an extensive review of the selected articles, it was found that 10 of the 21 articles focused on detecting IH, and 15 articles utilized AI. However, the best-performing technique in detecting IH employed DI. Additionally, the SLR offers insights and recommendations into future directions for IH applications.

## 1. Introduction

The most common type of benign tumor in infants is called infantile hemangioma (IH). It affects around 3–10% of preterm newborns [1]. It usually surfaces around the first few weeks of life and enlarges quickly throughout the first six months [2], and, in most cases, it begins to become smaller and goes away entirely by the time a child is five or ten years old. However, in some cases, an IH might cause complications as it comprises a group of abnormal blood vessels that can vary in shape, size, and color. IH lesions can be red, bluish, or even purplish in color. Depending on where they are, they can occasionally result in consequences including bleeding, ulceration, or problems with breathing, feeding, or eyesight. The size, location, and intensity of the lesion are among the characteristics that determine the treatment options for infantile hemangiomas. Treatment is rarely required because the hemangioma will usually go away on its own with time. However, if action is required, the choices include drugs, laser treatment, or, in extreme circumstances, surgical excision.

Early diagnosis of IH enables medical professionals to monitor the growth of hemangiomas, take action to stop complications like breathing problems or vision impairment, and provide the appropriate management techniques [3]. Early detection also makes it possible for medical professionals to support families emotionally as they work through the difficulties brought on by the illness. In general, impacted individuals’ quality of life is improved and their results are improved with early recognition.

Also, as hemangiomas vary in shapes and sizes from patient to patient, precise techniques to measure and predict their evolution are required for proper treatment. Currently, the measurement of IH lesions is conducted manually by professionals using a measuring tape to record the height and width of the lesions [4]. However, since these lesions are usually irregular in shape, the conventional method is not precise as it does not convey any information about the geometry of the lesions. Therefore, the automatic segmentation of the IH lesions plays an important role in the treatment process.

After detecting and segmenting IH lesions, the treatment process starts. As the patient starts receiving the proper treatment, the lesions start to diminish. However, there are no standard guidelines to evaluate the treatment response of IH [5]. As a result, the automatic evaluation of the treatment response is another important field in the literature.

In this paper, a systematic literature review (SLR) is performed on the techniques in the literature concerned with IH using artificial intelligence (AI) and digital imaging techniques. Papers conducted from 2014 to 2024 (April) are included in this work. The rest of the paper is divided as follows: the related surveys and reviews implemented regarding IH are mentioned in Section 2. The methodology followed to conduct the SLR is described in Section 3, while Section 4 provides the answers to the research questions and the discussion. Finally, the conclusions, limitations, and future work are discussed in Section 5.

## 2. Literature Review

Yang et al. [6] conducted a survey on the use of AI applications in pediatric oncology diagnosis, where they focused on different types of tumors, including infantile hemangioma (IH). The authors concluded that the lack of sufficient medical datasets is restraining AI from being an effective diagnostic tool in pediatric oncology. However, they predicted that the use of data augmentation techniques and detailed segmentation along AI can result in satisfactory results.

Another survey on AI applications to pediatric dermatology was performed by Burshtein and Buethe [7]. They mentioned that the majority of the research on AI and dermatological disorders focuses on skin malignancies, both melanoma and non-melanoma. Psoriasis, acne vulgaris, onychomycosis, and atopic dermatitis are other disorders that are often researched and have differing degrees of accuracy, sensitivity, and specificity. They also stated that AI has emerged recently in diagnosing IH.

Wang et al. [8] conducted a comprehensive bibliometric analysis on the global research on applications of AI in dermatology, stating that it is critical to diagnose infantile hemangiomas timely and accurately to avoid any potential adverse effects.

This work differs from other surveys as it focuses solely on the use of AI and digital imaging techniques regarding IH since the prior works only considered IH as part of their research and only referenced a few papers in that regard.

## 3. Materials and Methods

**Figure 1:**
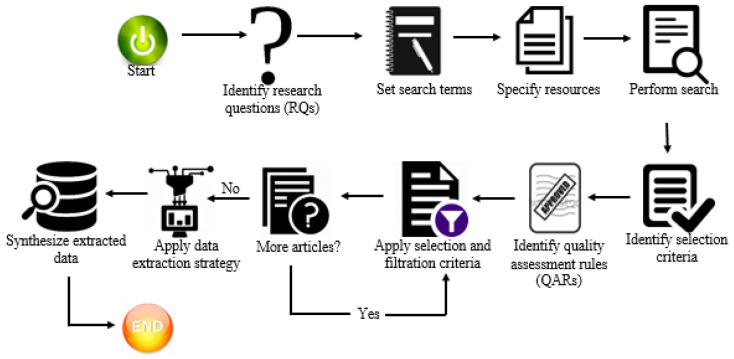
The overall process of Kitchenham framework [10].

The systematic literature review (SLR) is conducted by following Kitchenham and Charters’ framework [9]. Kitchenham and Charters’ SLR methodology includes three phases, which are planning, conducting, and reporting, and each phase contains several stages. Figure 1 represents the overall process.

### 3.1. Planning Phase

The first part of the planning phase is to define the objective of the SLR. The main goal of this SLR is to comprehensively evaluate and synthesize the existing research on the utilization of digital imaging techniques and artificial intelligence (AI) in different areas of infantile hemangioma. This review aims to provide a comprehensive overview of the current state of knowledge, identify gaps in the literature, and offer insights for future research and clinical practice in the field. Therefore, the following research questions are to be answered:

1. **RQ1:** What are the issues solved using AI and digital imaging in infantile hemangioma?
2. **RQ2:** What are the most-used AI and digital imaging techniques in IH?
3. **RQ3:** Which technique provided the best performance in IH detection?
4. **RQ4:** What are the limitations and future directions in the various fields of IH utilizing AI and digital imaging?

The search terms used to obtain the search results are as follows:

- “Artificial Intelligence” OR “AI” AND “Detection” OR “Segmentation” OR “Classification” OR “Treatment response” OR “Diagnosis” AND “Infantile Hemangioma” OR “IH”;
- “Digital imaging” AND “Detection” OR “Segmentation” OR “Classification” OR “Treatment response” OR “Diagnosis” AND “Infantile Hemangioma” OR “IH”;
- “Color” AND “Image” AND “Detection” OR “Segmentation” OR “Classification” OR “Treatment response” OR “Diagnosis” AND “Infantile Hemangioma” OR “IH”.

The digital libraries used to specify and collect the related research papers are listed below:

- Google Scholar;
- IEEE Explorer;
- Elsevier;
- Springer;
- MDPI.

### 3.2. Conducting Phase

**Figure 2:**
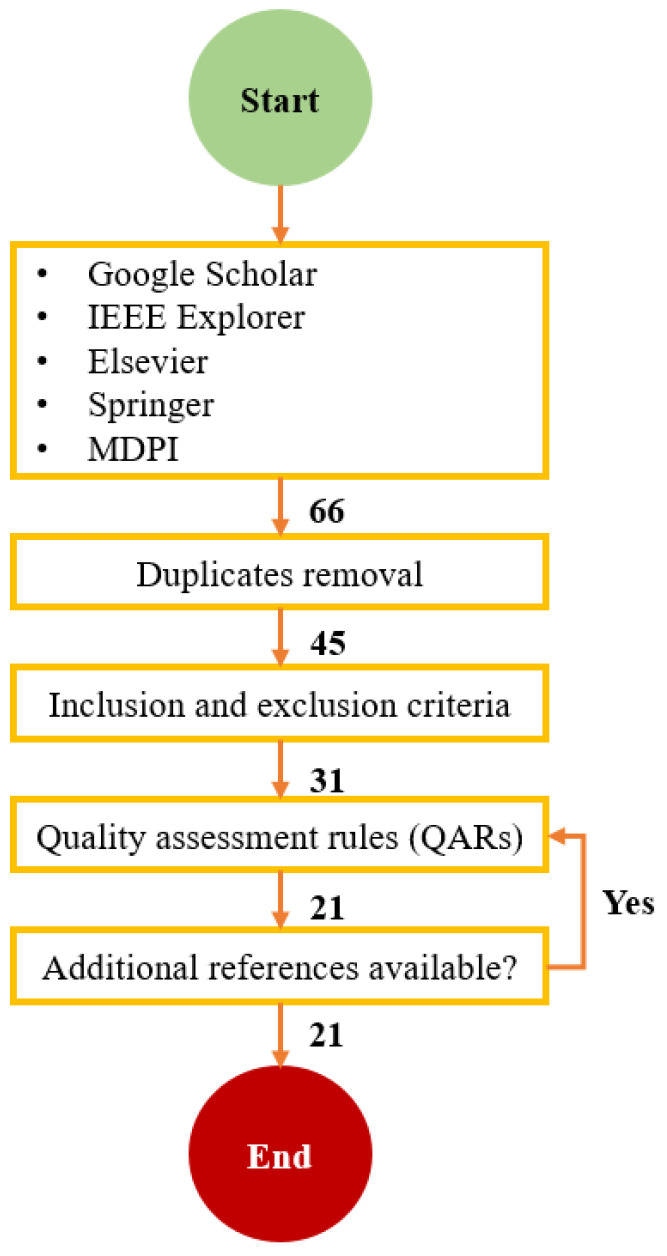
Filtration process of collected research papers.

The second phase in the SLR is conducting phase; it includes five stages. It starts with research identification, then selection study in order to select related studies based on specific criteria, after that quality evaluation by applying Quality Assessment Rules, then extracting data by studying the selected and filtered research, and finally synthesize the collected data. A total of 41 research papers were collected using the search terms. Then, the filtration technique from [10] is used, as shown in Figure 2.

The inclusion and exclusion criteria used to select the studies are listed below.

**Inclusion criteria:**

- Consider papers that utilize AI to detect/classify/segment/evaluate the treatment of IH.
- Consider papers that utilize digital imaging to detect/classify/segment/evaluate the treatment of IH.
- Consider studies that compare between different AI techniques that detect/classify/segment/evaluate the treatment of IH.
- Consider studies that compare between different digital imaging techniques that detect/classify/segment/evaluate the treatment of IH.
- Select the last update and edition of the articles.
- Select research papers published between January 2014 and April 2024.

**Exclusion criteria:**

- Exclude research papers that include detection/classification/segmentation/evaluation of the treatment of IH that are not related to AI.
- Exclude research papers that include detection/classification/segmentation/evaluation of the treatment of IH that are not related to digital imaging.
- Eliminate papers that are not journals and conferences.
- Eliminate journal papers that are not Q1 or Q2.

After the filtration steps, 21 research papers are selected for this SLR. These articles are recorded in Table A1 in Appendix A.

To assess the adequacy of the selected papers in regard to the research questions, Quality Assessment Rules (QARs) were utilized. Each QAR has 1 mark if fully answered (100% answered = 1), answered above the average (75% answered = 0.75), average 0.5 (50% answered = 0.5), answered below the average 0.25 (25% answered = 0.25), and, if the paper did not answer the QARs, the mark = 0.

1. **QAR 1:** Is the paper well organized?
2. **QAR 2:** Are the research objectives identified clearly in the paper?
3. **QAR 3:** Is there sufficient background information provided in the paper?
4. **QAR 4:** Is the conducted experiment suitable and acceptable?
5. **QAR 5:** Are the results of the conducted experiments clearly identified and reported?

The addition of each QAR score results in the total score for each paper; the selected papers need to have a total score of at least three out of five. The scores in detail for each paper as well as the total score are listed in Table A2 in Appendix A.

After selecting the 21 research papers that passed the QARs, data extraction is performed on them to extract the needed information to answer the research questions identified for the SLR. The extracted data include the source (conference/journal), year of publication, title, type of application (detection/classification/segmentation/treatment evaluation), technique used, methodology, dataset information (number of classes/dataset size), results, limitations, and future work.

Finally, after extracting the data, quantitative and qualitative procedures are used to synthesize them. The first research question is narrative, which represents the issues solved in infantile hemangioma using AI or digital imaging. The second research question is qualitative, which is about the most frequently used algorithms. The third question is quantitative since it is concerned with the best-performing technique. The last research question is also narrative since it extracts the limitations and future direction in the field of IH using AI and digital imaging.

## 4. Results and Discussion

**Figure 3:**
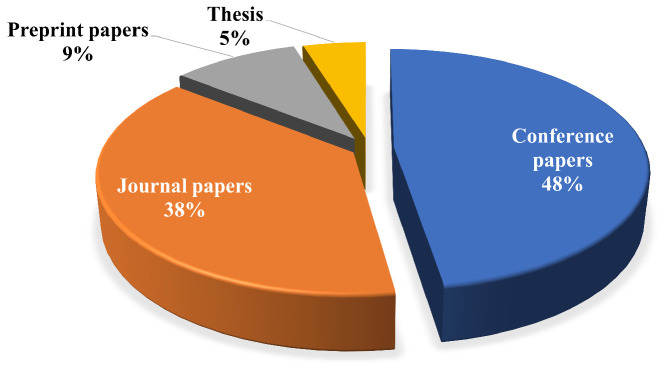
Distribution of collected papers.

In the final phase (the reporting phase), the research questions are answered based on the synthesized and extracted data from the 21 collected articles. Figure 3 displays a pie chart that provides the distribution of the collected articles in terms of conference, journal, preprint, and thesis papers. It is clear from the chart that the majority of the papers are journals, followed by conferences, then preprints, and finally one thesis report.

### 4.1. The Infantile Hemangioma Problems Solved in the Selected Research Papers (RQ1)

Since IH could lead to major complications in infants if it is not detected and treated, many researchers have utilized AI-based algorithms to automatically detect and classify IH. Some papers classified the images into two classes: IH and non-IH, or IH and another disease [11,12,13,14,15], while others classified the images into three or more classes, including other dermatology diseases [16,17,18,19,20]. In addition, some articles did not specify the number of classes [21,22].

Another issue encountered by researchers in IH utilizing AI and digital imaging is the segmentation of IH lesions. In order to properly treat an IH lesion, precise measurements need to be taken of it. Consequently, Refs. [4,23,24,25] applied AI and digital imaging techniques to segment the images into two classes: IH and non-IH. In addition, Oprisescu et al. [4] performed measurements of the lesions following segmentation, where they enhanced their work in [23] by adding geometric correction to preprocess the images to look like the lesions are perpendicular to the camera and that the images are taken from a constant distance from the camera, which ensures accurate measurements of the lesions.

After receiving a treatment for IH, tracking the response of the treatment is beneficial to ensure that the treatment is indeed working. Therefore, some researchers [5,26,27,28] developed algorithms to assess the treatment response in follow-up appointments.

Finally, the most understudied area in terms of the collected papers is risk rating of IH lesions. Based on numerous factors, specialized doctors classify IH lesions into three categories: high, medium, and low risk. These factors include the child’s age, lesion depth, size, location, and growth rate, as well as other factors. Chen and Fu [29] proposed a Deep Learning method to automatically rate the risk of IH lesions.

**Figure 4:**
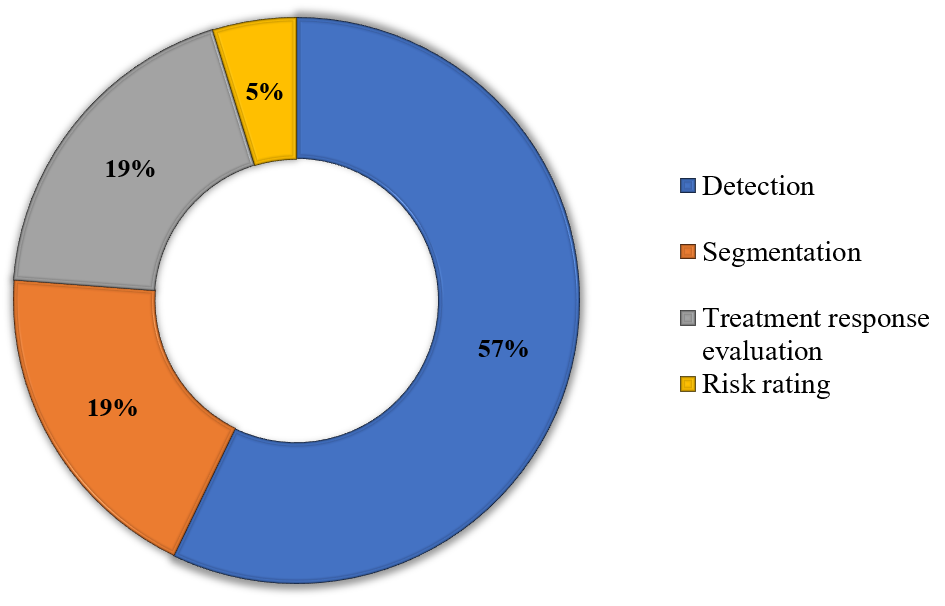
Doughnut chart of different problems solved by the selected articles.

Figure 4 presents a doughnut chart specifying the percentages of each area in IH that has been tackled by the collected articles. It is clear that detection of IH is the most practiced area, holding a percentage of 57%, followed by segmentation of IH lesions and treatment response evaluation at 19% each, and risk rating is the least-practiced field with a percentage of 5%.

### 4.2. The Most-Used AI and Digital Imaging Techniques in Infantile Hemangioma (RQ2)

**Figure 5:**
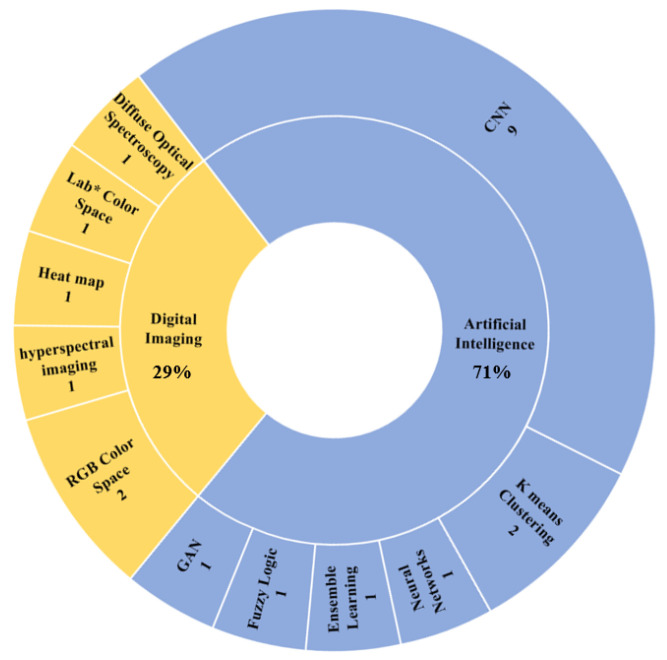
Sunburst chart of different techniques utilized by the selected articles. The numbers below every technique represent the number of selected articles that employ them.

Artificial intelligence (AI) is the simulation of human intelligence processes by machines. It is divided into Machine Learning (ML) and Deep Learning (DL). As can be seen from Figure 5, the AI-based techniques utilized in IH represent 71% of the selected papers, where most of these employ Convolutional Neural Networks (CNNs). CNNs are a subcategory of DL where convolution is used to detect patterns in the input image. CNNs are designed to process structured data such as images, which justifies their application as the most-used technique because all IH applications depend heavily on patient images.

On the other hand, the digital imaging technology used in IH represents 29% of the selected articles found, as displayed in Figure 5. The RGB color space is employed by the majority of those papers utilizing digital imaging. The RGB color space [30] is a three-dimensional representation of color, those three dimensions being red, green, and blue. The intensities of each of these colors range from 0 to 255. By mixing the three primary colors, a wide range of colors are produced. Ref. [13] conducted a multi-center analysis on the RGB and HSL (hue, saturation, and lightness) values of IH lesions or port-wine birth marks. Another study was conducted by [28] to find the RGB ratio and differences between lesions and normal tissue.

### 4.3. The Best-Performing Infantile Hemangioma Detection Technique (RQ3)

To perform a fair comparison between the techniques, they need to have some common ground. However, this is not the case in IH applications since datasets are scarce and researchers mostly use private datasets obtained by their local hospitals. Therefore, for the detection of IH, only papers that aimed to detect two classes, IH and non-IH, were compared. Table 1 summarizes the performance of the selected papers along with the used dataset sizes. The following metrics were used: accuracy, recall/sensitivity, and specificity [31]. The metrics are defined in Equations (1)–(3), where TP is defined as true positive, TN as true negative, FP as false positive, and FN as false negative.

(1) $$Accuracy=\frac{TP+TN}{TP+TN+FP+FN}$$

(2) $$Recall=\frac{TP}{TP+FN}$$

(3) $$Specificity=\frac{TN}{FP+TN}$$

Accuracy is an important metric to consider; however, in the case of class imbalance, it is not the best representation of the model performance. On the other hand, recall is highly important. Infantile hemangiomas, especially if left untreated or undetected, can lead to complications or require medical intervention. Ensuring that your model has high recall means minimizing the chances of missing any actual cases of infantile hemangiomas (i.e., false negatives). Therefore, maximizing recall helps to ensure that all the instances of infantile hemangiomas are detected, even if it leads to some false positives. Finally, specificity is also an important metric in this scenario since false positives (incorrectly classifying non-hemangioma images as hemangiomas) can lead to misdiagnoses of other skin lesions where the patient could receive the wrong treatment, which could in turn lead to complications depending on the type of lesion. Moreover, specificity is key to minimizing unnecessary treatments or interventions for cases that are not actually infantile hemangiomas. Therefore, by comparing the performances in Table 1, we conclude that A11 provides the best detection performance in a collective manner regarding the three metrics.

### 4.4. Limitations and Future Directions in IH Utilizing AI and Digital Imaging (RQ4)

The limitations for all the selected articles are summarized in Table 2. By addressing the limitations in Table 2 and the current trends in the research, various future directions can be suggested. The dataset size and quality are highly important to ensure good performance of any technique. Therefore, a large benchmark dataset for infantile hemangioma needs to be collected in consistent conditions and properly labeled by specialized doctors. In addition, multimodal imaging can be applied to IH applications such as ultrasound or MRI to provide complementary information and a more comprehensive evaluation of the hemangioma characteristics. Also, integrating other relevant parameters such as skin texture, lesion volume, and possibly 3D imaging techniques could lead to improved performance in IH applications. Another possible direction is exploring methods to enhance the interpretability and explainability of AI models.

## 5. Conclusions

This systematic literature review investigated the utilization of artificial intelligence and digital imaging in infantile hemangioma (IH) applications, with the objective of addressing four key research inquiries. The findings revealed that IH detection emerged as the most prevalent focus in the literature, with Convolutional Neural Networks (CNNs) being the predominant technique employed in the IH applications. Among the various methods evaluated, CNNs exhibited superior performance in terms of accuracy, recall, and specificity for IH detection. This early detection can help to prevent severe complications in infants. In addition, AI and digital imaging techniques offer the precise segmentation of IH lesions, which is crucial for measuring and treating these irregularly shaped lesions. This improves the treatment process by providing accurate information. Moreover, AI algorithms are capable of assessing the treatment response in IH patients, helping medical professionals to track the effectiveness of the treatment over time. Finally, DL techniques have been applied to classify IH lesions based on risk factors such as the child’s age, lesion depth, size, and location, assisting in determining the urgency of treatment.

Furthermore, the review identified the limitations within the selected studies. A significant limitation in the application of AI models to medical imaging is the lack of large, standardized, and publicly available datasets, which restricts both the model performance and the generalizability. Many studies also encounter challenges with dataset imbalance, where certain classes are underrepresented, leading to biased outcomes. Additionally, the quality of the images used for training can be compromised by factors such as infant movement and inconsistent lighting, affecting the accuracy of the detection and segmentation. Techniques like Generative Adversarial Networks (GANs) require substantial computational resources, creating barriers to their adoption in clinical settings. The absence of standardized imaging protocols and evaluation guidelines further complicates the comparison of results across different studies and hinders the consistent application of AI solutions. Moreover, the “black box” nature of many Deep Learning models, where the decision-making processes are not easily interpretable, poses a challenge in medical applications where explainability is crucial.

Based on this review, we recommend researchers to focus on collaborating across multiple medical institutions to collect large and comprehensive multimodal datasets for IH lesions with different shapes, sizes, colors, and locations that are adequately labeled by specialized doctors. Another recommendation is to implement standardized imaging protocols, such as consistent lighting, standardized camera settings, and clear guidelines for capturing images, to reduce variability and improve image quality. We also recommend using interpretable AI to provide an explainable performance that enhances the understanding of professional doctors in detecting and treating IH. Lastly, researchers are also recommended to focus mainly on increasing sensitivity (recall) and specificity instead of only accuracy as they hold more significance in medical applications.

## Figures and Tables

**Table 1:** Comparison between the performance of IH detection techniques.

Article ID	Technique	Preprocessing/Methodology	Dataset Size	Accuracy	Recall	Specificity
**A5**	CNN	Resize and crop	240	**93.84%**	91.45%	**96.24%**
**A6**	CNN	Flip, rotate, Lab* colorspace (used normalized a layer)	80	91%	92%	90%
**A11**	Electronic Colorimetries	Analysis of RGB and HSL values	176	90%	92%	**98%**
**A17**	CNN	Pretrained Resnet-50 architecture, random oversampling to balance dataset	5843	91.7%	**93%**	90.5%

**Table 2:** Limitations of selected articles.

Application	ID	Limitations
**Detection**	A4	Dataset imbalance.
	A5	Poor quality of images due to infant movements and different lighting conditions; small dataset.
	A6	Small dataset size.
	A8	Small dataset size.
	A10	Training Generative Adversarial Networks (GANs) requires significant computational resources.
	A11	Dataset used composed of infants less than 3 months old, so the generalizability of the results to older infants was not addressed.
	A17	Imbalanced dataset; random oversampling may lead to model overfitting.
	A18	Poor quality of images due to infant movements.
	A19	Imbalanced dataset.
	A21	The system’s limitation in tissue penetration depth, up to a few millimeters, may restrict its applicability for deeper tissue analysis.
**Segmentation**	A1	Small dataset size; lack of standardized image acquisition protocol; need for a robust evaluation protocol.
	A3	Error analysis: calculation of the Haussdorf distance suggested that the shortest path method made fewer strong errors in comparison to CNN, albeit potentially making more errors overall.
	A12	Inconsistent imaging conditions due to large time period of collection.
	A15	Small dataset size.
**Treatment** **response** **evaluation**	A2	Ocular protection is needed to use the proposed device on lesions closer to the eye.
	A7	Small dataset size; standardized reference patch use leads to challenges in clinical practice.
	A20	The duration of the follow-up period in the study may have been relatively short, which could impact the ability to assess long-term treatment outcomes and the sustainability of the proposed digital imaging system.

## Data Availability

No new data were created or analyzed in this study. Data sharing is not applicable to this article.

## Abbreviations

AI	Artificial Intelligence
CNNs	Convolutional Neural Networks
DL	Deep Learning
GANs	Generative Adversarial Networks
HSL	Hue, Saturation, Lightness
IH	Infantile Hemangioma
ML	Machine Learning
QARs	Quality Assessment Rules
RGB	Red, Green, Blue
RQ	Research Question
SLR	Systematic Literature Review

## Appendix A

Table A1 summarizes the selected articles included in the systematic literature review. Table A2 displays the scores for the Qualtiy Assesment Rules questions for each of the selected articles. A total score of 5 is the maximum. Articles with less than three out of five are not included in the SLR. Table A3 summarizes the details of the slected articles in terms of application, used technique, dataset size, number of classes and patients, and age. NA stands for not available.

**Table A1:** The titles of the selected articles in the systematic literature review.

Article ID	Article Title	Year	Ref.
**A1**	Automatic Measurement of Infantile Hemangiomas	2017	[23]
**A2**	Assessment of Infantile Hemangiomas Using a Handheld Wireless Diffuse Optical Spectroscopic Device	2017	[26]
**A3**	The Challenges of Applying Deep Learning for Hemangioma Lesion Segmentation	2018	[24]
**A4**	Identifying Pediatric Vascular Anomalies With Deep Learning	2019	[16]
**A5**	Infantile Hemangioma Detection using Deep Learning	2020	[11]
**A6**	Automatic Detection of Infantile Hemangioma using Convolutional Neural Network Approach	2020	[12]
**A7**	Colorimetric analysis of images in the follow-up of infantile hemangiomas	2020	[5]
**A8**	The Application of Deep Learning Algorithms to Pediatric Vascular Anomalies Using a Limited Dataset	2021	[17]
**A9**	Risk Rating of Infantile Hemangioma using Deep Learning	2021	[29]
**A10**	An Infantile Hemangioma Dataset IH-2021 and a Deep Learning based Recognition Method on it	2021	[21]
**A11**	Analysis of lesional color to differentiate infantile hemangiomas from port-wine birthmarks in infants less than 3 months old: A pilot study	2021	[13]
**A12**	Automatic Segmentation and Measurement of Infantile Hemangioma	2021	[4]
**A13**	Mapping of Segmental and Partial Segmental Infantile Hemangiomas of the Face and Scalp	2021	[27]
**A14**	Intelligent Diagnosis of Vascular Anomalies with Deep Learning	2022	[19]
**A15**	Infantile Hemangiomas Segmentation using U-Net and SegNet Architectures Models	2022	[25]
**A16**	Autonomous diagnosis of pediatric cutaneous vascular anomalies using a convolutional neural network	2022	[18]
**A17**	Development of an artificial intelligence algorithm for the diagnosis of infantile hemangiomas	2022	[14]
**A18**	Infantile hemangiomas evaluation based on hyperspectral imaging	2023	[15]
**A19**	Ensemble of Self-supervised Learning Methods for Robust Skin Disease Image Diagnosis Leveraging Unlabeled Data	2023	[20]
**A20**	Development of a digital imaging analysis system to evaluate the treatment response in superficial infantile hemangiomas	2023	[28]
**A21**	Rapid low-cost hyperspectral imaging system for quantitative assessment of infantile hemangioma	2023	[22]

**Table A2:** The Quality Assessment Rules scoring for the selected articles in the systematic literature review.

Article ID	QAR1	QAR2	QAR3	QAR4	QAR5	Total Score
**A1**	1	0.5	1	0.5	1	4
**A2**	1	1	1	0.5	0.5	4
**A3**	1	0.5	1	1	1	4.5
**A4**	1	0.5	1	1	1	4.5
**A5**	0.5	1	1	1	1	4.5
**A6**	1	0.5	1	0.5	1	4
**A7**	1	1	1	0.5	0.5	4
**A8**	1	1	1	1	1	5
**A9**	1	0.5	1	1	1	4.5
**A10**	1	0.5	1	1	1	4.5
**A11**	0.5	1	1	1	1	4.5
**A12**	1	1	1	0.5	0.5	4
**A13**	1	1	1	0.5	0.5	4
**A14**	1	0.5	1	0.5	0.5	3.5
**A15**	0.5	1	1	1	1	4.5
**A16**	1	1	1	1	0.5	4.5
**A17**	1	0.5	1	1	1	4.5
**A18**	1	1	0.5	0.5	0.5	3.5
**A19**	1	1	1	0.5	1	4.5
**A20**	1	1	1	0.5	1	4.5
**A21**	1	0.5	1	0.5	1	4

**Table A3:** Details of selected articles.

Article ID	Application	Technique	Dataset Size	# of Classes	# of Patients	Age
**A1**	Segmentation	ML + Image processing	20	2	NA	NA
**A2**	Evaluate treatment response + classification	Image processing	15 IH cases	3	13	5–7 months
**A3**	Segmentation	CNN	200	2	29	NA
**A4**	Detection	CNN	21,681	6 and 12	NA	4 months
**A5**	Detection	CNN	240	2	40	NA
**A6**	Detection	CNN + Fuzzy c-means and Otsu method	80	2	25	NA
**A7**	Evaluate treatment response	Image processing	17	2	17	1–10 months
**A8**	Detection	CNN	373	5	NA	NA
**A9**	Risk Rating	CNN	1032	3	344	NA
**A10**	Detection	Generative Adversarial Networks + ResNet50	2995	2	NA	NA
**A11**	Detection	Image processing	176	2	176	Less than 3 months
**A12**	Segmentation	ML + Image processing	30	2	NA	NA
**A13**	Evaluate treatment response	Image processing	549	4	NA	Less than 12 years
**A14**	Detection	CNN	963	3	529	NA
**A15**	Segmentation	CNN (U-Net and SegNet)	220	2	35	NA
**A16**	Detection	CNN	930	5	NA	0–16 months
**A17**	Detection	CNN	5834	2	1421	0–1 years
**A18**	Detection	Digital Imaging + DL	NA	2	2	Under 6 months
**A19**	Detection	Ensemble approach	10,000	198	NA	NA
**A20**	Evaluate treatment response	Image processing	207	NA	207	3–5 months
**A21**	Detection	Image processing	NA	NA	6	2 weeks–12 months

## References

[1] Haggstrom A.N., Drolet B.A., Baselga E., Chamlin S.L., Garzon M.C., Horii K.A., Lucky A.W., Mancini A.J., Metry D.W., Newell B. (2007). Prospective study of infantile hemangiomas: Demographic, prenatal, and perinatal characteristics. J. Pediatr..

[2] Darrow D.H., Greene A.K., Mancini A.J., Nopper A.J., Antaya R.J., Cohen B., Drolet B.A., Fay A., Fishman S.J., Friedlander S.F. (2015). Diagnosis and management of infantile hemangioma. Pediatrics.

[3] Holland K.E., Drolet B.A. (2010). Infantile hemangioma. Pediatr. Clin..

[4] Oprisescu S., Ciuc M., Sultana A. (2021). Automatic Segmentation and Measurement of Infantile Hemangioma. Symmetry.

[5] Abagge K.T., Sandri C.d.O., Sakai L.S.M., Mussato L.P., Petterle R.R., Oliveira de Carvalho V.O. (2020). Colorimetric analysis of images in the follow-up of infantile hemangiomas. Pediatr. Dermatol..

[6] Yang Y., Zhang Y., Li Y. (2023). Artificial intelligence applications in pediatric oncology diagnosis. Explor. Target. Anti-Tumor Ther..

[7] Burshtein J., Buethe M.G. (2024). Artificial Intelligence in Dermatology: A Review of Literature and Application to Pediatric Dermatology. SKIN J. Cutan. Med..

[8] Wang G., Meng X., Zhang F. (2023). Past, present, and future of global research on artificial intelligence applications in dermatology: A bibliometric analysis. Medicine.

[9] Keele S. (2007). Guidelines for Performing Systematic Literature Reviews in Software Engineering.

[10] Injadat M., Salo F., Nassif A.B. (2016). Data mining techniques in social media: A survey. Neurocomputing.

[11] Sultana A., Balazs H., Ovreiu S., Oprisescu S., Neghina C. Infantile hemangioma detection using deep learning. Proceedings of the 2020 13th International Conference on Communications (COMM).

[12] Horvath B., Neghina C., Griparis A., Sultana A. Automatic Detection of Infantile Hemangioma using Convolutional Neural Network Approach. Proceedings of the 2020 International Conference on e-Health and Bioengineering (EHB).

[13] O’Brien K.F., Frieden I.J., Zeymo A., Vasic J., Silverman R., Goldberg G., Carver DeKlotz C.M. (2021). Analysis of lesional color to differentiate infantile hemangiomas from port-wine birthmarks in infants less than 3 months old: A pilot study. Pediatr. Dermatol..

[14] Zhang A.J., Lindberg N., Chamlin S.L., Haggstrom A.N., Mancini A.J., Siegel D.H., Drolet B.A. (2022). Development of an artificial intelligence algorithm for the diagnosis of infantile hemangiomas. Pediatr. Dermatol..

[15] Shupletsov V., Gorunov I., Sergienko M., Zhurilo I., Potapova E., Dremin V. Infantile hemangiomas evaluation based on hyperspectral imaging. Proceedings of the European Conference on Biomedical Optics.

[16] Chan J., Raju S., Bly R., Perkins J.A., Gollakota S. (2019). Identifying Pediatric Vascular Anomalies With Deep Learning. arXiv.

[17] Robertson B.I. (2021). The Application of Deep Learning Algorithms to Pediatric Vascular Anomalies Using a Limited Dataset. Bachelor’s Thesis.

[18] Patel P., Ragland K., Robertson B., Ragusa G., Wiley C., Miller J., Jullens R., Dunham M., Richter G. (2022). Autonomous diagnosis of pediatric cutaneous vascular anomalies using a convolutional neural network. Int. J. Pediatr. Otorhinolaryngol..

[19] Cai Y., Gong X., He Q., Fan X., Xiong P. Intelligent Diagnosis of Vascular Anomalies with Deep Learning. Proceedings of the 3rd International Symposium on Artificial Intelligence for Medicine Sciences.

[20] Kojima K., Tadokoro R., Kinoshita K., Asano Y., Yamasaki K., Shido K. (2023). Ensemble of Self-supervised Learning Methods for Robust Skin Disease Image Diagnosis Leveraging Unlabeled Data. Res. Sq..

[21] Zhang X., Gao L., Li L., Yan Z., Yu L. An Infantile Hemangioma Dataset IH-2021 and a Deep Learning based Recognition Method on it. Proceedings of the 2021 IEEE International Conference on Bioinformatics and Biomedicine (BIBM).

[22] Perkov S., Vorobev V., Kurochkin M.A., Gorodkov S., Gorin D. (2024). Rapid low-cost hyperspectral imaging system for quantitative assessment of infantile hemangioma. J. Biophotonics.

[23] Oprisescu S., Ciuc M., Sultana A. Automatic measurement of infantile hemangiomas. Proceedings of the 2017 E-Health and Bioengineering Conference (EHB).

[24] Alves P.G., Cardoso J.S., do Bom-Sucesso M. The challenges of applying deep learning for hemangioma lesion segmentation. Proceedings of the 2018 7th European Workshop on Visual Information Processing (EUVIP).

[25] Sultana A.E., Oniga M., Nitu C., Sandu C.N., Petrescu A.S. Infantile Hemangiomas Segmentation using U-Net and SegNet Architectures Models. Proceedings of the 2022 E-Health and Bioengineering Conference (EHB).

[26] Fong C.J., Garzon M.C., Hoi J.W., Kim H.K., Lauren C.T., Morel K., Geller L., Antonov N., Weitz N., Wu J. (2017). Assessment of infantile hemangiomas using a handheld wireless diffuse optical spectroscopic device. Pediatr. Dermatol..

[27] Endicott A.A., Chamlin S.L., Drolet B.A., Mancini A.J., Siegel D.H., Vitcov S., Mathes E.F., Frieden I.J., Haggstrom A.N. (2021). Mapping of segmental and partial segmental infantile hemangiomas of the face and scalp. JAMA Dermatol..

[28] Xie M., Liu J., Zhou P., Xu X., Liu H., Zeng L., Chen F., Zeng Y., Huang H., Peng W. (2023). Development of a digital imaging analysis system to evaluate the treatment response in superficial infantile hemangiomas. PLoS ONE.

[29] Chen B., Fu G. Risk Rating of Infantile Hemangioma using Deep Learning. Proceedings of the 2021 2nd International Symposium on Computer Engineering and Intelligent Communications (ISCEIC).

[30] Süsstrunk S., Buckley R., Swen S. Standard RGB color spaces. Proceedings of the Color and Imaging Conference.

[31] Buckland M., Gey F. (1994). The relationship between recall and precision. J. Am. Soc. Inf. Sci..

